# Impact of N-1 phase media optimization as a bioprocess intensification strategy on fed-batch production

**DOI:** 10.1007/s00449-026-03370-2

**Published:** 2026-06-25

**Authors:** Mustafa Doğukan Metiner, Elif Damla Arisan, Abdullah Uslu, Işıl Kurnaz

**Affiliations:** 1 Institute of Biotechnology, Gebze Technical University, 41400 Gebze, Kocaeli Türkiye; 2 Marmara Teknokent (MARTEK) Biotechnology R&D Center, Nobel Pharmaceuticals, 41400 Gebze, Kocaeli Türkiye; 3 Dept of Molecular Biology and Genetics, Gebze Technical University, 41400 Gebze, Kocaeli Türkiye

**Keywords:** Bioprocess optimization, CHO-ZN cell line, High cell density, N-1 intensification, Process efficiency

## Abstract

In the biopharmaceutical industry, improving process efficiency and reducing manufacturing costs remain major priorities. Conventional fed-batch processes are often limited by low initial cell densities, resulting in extended culture duration and reduced productivity. However, the combined effect of N-1 media optimization and high-cell-density (HCD) inoculation remains insufficiently characterized. This study systematically assessed the impacts of N-1 media optimization and initial seeding density utilizing a CHO-ZN cell line. During the N-1 phase, a media exchange strategy increased the cell density from approximately 3 × 10⁶ cells/mL to 15–22 × 10⁶ cells/mL, enabling direct production inoculation at 8 × 10⁶ cells/mL without an additional seed expansion step. Compared with low-cell-density (LCD; 1.2 × 10⁶ cells/mL) cultures, HCD cultures reduced production duration by approximately 36% and increased product concentration by about 89% (~ 3500 mg/L vs. ~1850 mg/L). Average interval-based specific productivity (qP), increased from approximately 17 to 31 pg/cell/day, suggesting that the gains in productivity were not solely attributable to increased biomass. Importantly, when the production-phase medium was standardized, differences in titer concentration, metabolite profiles, viability, and interval-based qP persisted among cells conditioned in different N-1 media. This indicates that N-1 medium composition influenced subsequent fed-batch behavior not only by affecting inoculum biomass, but also by modulating the physiological state of the cells prior to production inoculation. Overall, these findings emphasize the role of N-1 medium-dependent physiological conditioning in determining productivity, metabolic behavior, and product quality attributes under intensified fed-batch conditions.

## Introduction

Biopharmaceuticals, particularly monoclonal antibodies (mAbs), have transformed the treatment of numerous diseases globally [1–3]. They comprise the most significant part of the biopharmaceutical sector, expected to be worth more than USD 700 billion by 2030 and over USD 850 billion by 2050 [4, 5]. Mammalian cell lines, especially CHO cells, are the industry standard for manufacturing biopharmaceutical products using recombinant DNA technology [6, 7]. The mAb market, utilizing the CHO-ZN cell line, a type of CHO cell, has garnered the most attention in intensified production procedures due to its high titer yield and adaptability [8]. Despite its growing popularity for bioprocess development, there is still a lack of peer-reviewed research on how the CHO-ZN cell line behaves in intensified culture conditions. To fulfill the growing demand and make products more accessible, the present production processes need to be made more efficient and sustainable [3]. Thus, as biopharmaceutical companies strive to meet the rising demand for treatments while reducing costs and space requirements, process intensification solutions have become a significant focus of upstream process development [7, 9].

**Fig. 1:**
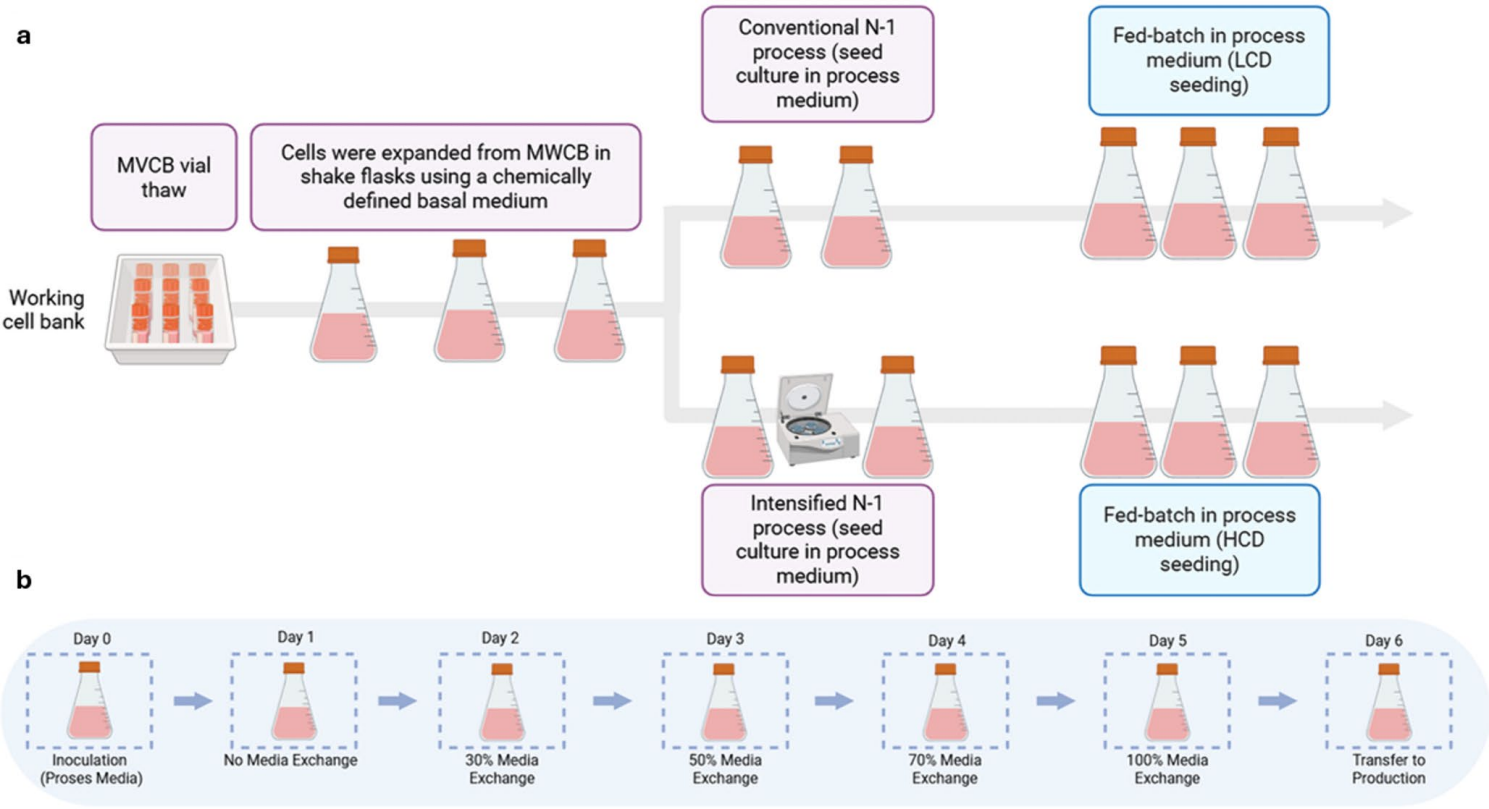
(a) Schematic representation of the cell expansion and seeding strategy. Two N-1 approaches were compared: a conventional N-1 process without medium exchange and an intensified N-1 process with stepwise medium exchange. (b) Schematic representation of the intensified N-1 medium-exchange progression (30–100%) and transfer to production on Day 6. Created with BioRender.com

Major limitations associated with conventional fed-batch bioprocesses include low cell densities and extended production durations [10–12]. In conventional fed-batch bioprocesses, the N-1 phase preceding production initiation is typically performed at relatively low cell densities (0.3–1.2 × 10⁶ cells/mL) (Fig. 1), which prolongs the overall culture duration and limits process throughput by reducing the number of fed-batch runs that can be completed within the same facility [13–15]. To overcome these limitations, N-1 process intensification strategies have been developed to accelerate cellular proliferation and enable high-seeding-density inoculation during the production phase [11, 12, 16]. In addition to increasing product yield, high-seeding-density strategies can shorten production duration and improve facility utilization [15]. Approaches such as perfusion, semi-perfusion, and medium exchange support enhanced cell viability and proliferation by reducing cellular stress, removing accumulated waste metabolites, and maintaining nutrient balance [2, 17]. In addition to perfusion-based intensification strategies, non-perfusion approaches using enriched batch or fed-batch seed cultures have also been reported to successfully achieve high inoculation densities and intensified fed-batch production performance [3]. Depending on the biopharmaceutical product and process configuration, N-1 intensification strategies can reduce process duration by 2–4 days while increasing product titers by 30–100%, thereby improving process efficiency, enhancing process productivity, and reducing production costs [14, 18–20]. Intensified N-1 strategies may improve production performance by reducing media consumption, shortening process duration, and decreasing operational waste generation [11, 21–25]. Biotechnological production facilities have a smaller environmental impact when they use less media and have shorter process times, resulting in less waste [26, 27]. These solutions enable the biopharmaceutical industry to meet its environmental sustainability goals [11, 28] by reducing water and energy consumption.

The primary objective of the N-1 stage optimizations is to enhance the productivity of the fed-batch process by facilitating the inoculation of production phase with elevated cell densities [28, 29]. Although non-perfusion intensified seed strategies have been reported, studies specifically investigating the impact of N-1 medium composition on subsequent production physiology and productivity remain limited [3]. However, there remains a lack of studies that systematically and quantitatively investigate the impact of targeted high cell densities in the N-1 stage on the production phase [10, 14, 30]. It has been demonstrated that reducing cellular stress, removing harmful metabolites, and restoring a balanced influx of new nutrients through media exchange during the N-1 stage promotes cell proliferation [10, 13]. However, for CHO-derived cell lines, the optimal timing, frequency, and volume ratios for implementing this strategy, particularly during specific growth phases, remain insufficiently specified [31, 32].

The primary objective of this study was to investigate how different N-1 culture media used to achieve elevated cell densities influence subsequent production yield and cell physiology under high-seeding-density fed-batch conditions. Nonetheless, in the pursuit of enhanced production outcomes, the effects of various N-1 media techniques on cell proliferation have not been systematically evaluated. To address this issue, we assessed cell growth kinetics at the N-1 stage using three different commercial medium compositions. Thereafter, cells grown under analogous conditions were used to initiate high-density fed-batch cultures, allowing for the comparison of monoclonal antibody titers, culture duration, and cell viability. Merck, which recommends a standard inoculum density of 1.2 × 10⁶ cells/mL for fed-batch processes, provided the CHO-ZN cell line used in this research. We commenced the production phase with a markedly increased seeding density of 8 × 10⁶ cells/mL to assess the effectiveness of process intensification strategies.

Although intensified seed-train strategies have been widely investigated, the extent to which N-1 medium composition modulates inoculum physiology and subsequently affects fed-batch productivity, metabolite accumulation, and product quality remains insufficiently defined. Therefore, this study evaluated three commercial N-1 media using a stepwise medium-exchange strategy and assessed their carry-over effects on high-seeding-density fed-batch production.

The findings demonstrate how N-1 stage optimization influences intensified fed-batch process performance. The CHO-ZN cell line demonstrated compatibility with high-density production conditions. These insights facilitate the logical design of upstream processes, improving the efficiency and productivity of monoclonal antibody production.

## Materials and methods

### Cell line and baseline processes

This research utilized a CHO-ZN cell line for the production of recombinant monoclonal IgG4 antibodies. This cell line was obtained (custom-generated) from Merck. Cells were initially cultured in 125 mL vented culture flasks containing EX-CELL^®^ CD CHO Fusion media (24365 C-10 L, Cytiva), which serves as Basal Expansion media, at a density of 0.4 × 10⁶ cells/mL (Fig. 1a). Subsequently, the cells were passaged every 3 days. During the N-1 stage, cells were switched to EX-CELL^®^ Advanced CHO Fed-batch medium (24365 C-10 L, Cytiva) (Process Medium) (Fig. 1a). On day 3, cells were inoculated into the production phase at a density of 1.2 × 10⁶ cells/mL (Fig. 1a). There was no media exchange throughout the fed-batch process; it was done in the same medium (Fig. 1a). From day 1 to day 4, LCD cultures were fed daily with 2% (v/v) Feed A (HyClone™ Cell Boost™ 7a, SH31026.02) and 0.2% (v/v) Feed B (HyClone™ Cell Boost™ 7b, SH31027.07) (Fig. 1b). From day 5 onward, the feeding ratios were adjusted to 2.5% (v/v) Feed A and 0.25% (v/v) Feed B and maintained until the end of the culture period.

We used these baseline process settings as a reference group to examine the impact of media exchange during the N-1 phase, which was applied at shake-flask scale as a simplified model to mimic key features of manufacturing-scale N-1 perfusion processes that typically use TFF- or ATF-based cell retention systems. All studies were conducted at flask scale in a regulated laboratory environment, maintained at temperature of 37 °C, a shaking velocity of 130 rpm, 25 mm orbit diameter, 85% humidity and a CO₂ concentration of 5%.

**Fig. 2:**
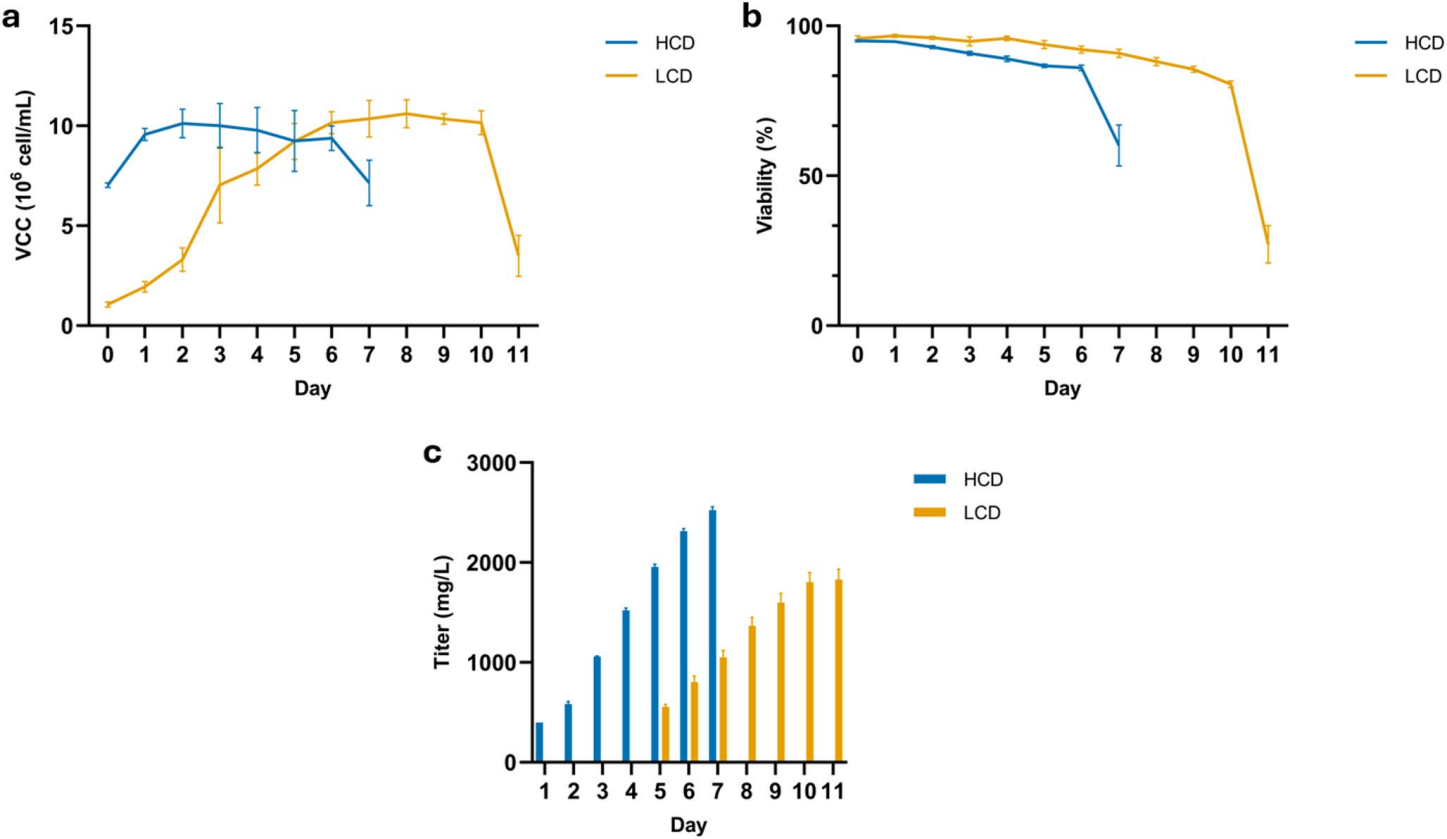
Changes in viable cell concentration (VCC), viability (%), and titer (mg/L) over time for CHO cultures that started with low (LCD) and high (HCD) seeding densities. (a) VCC profiles show faster growth and an earlier plateau in HCD cultures (blue) compared to LCD cultures (yellow). (b) Trends in viability over 11 days of cultivation. (c) Titer kinetics showing faster and higher mAb accumulation in HCD cultures compared with LCD cultures. Data are expressed as mean ± standard error of the mean (SEM) (*n* = 4)

To study the effect of initial cell density, the LCD group was seeded at 1.2 × 10^6^ cells/mL, while the test groups were seeded at 8 × 10^6^ cells/mL that was achieved by the N-1 intensification and cell concentration strategy explained. From day 1, the high-density groups were fed 4% Feed A and 0.4% Feed B every day. The titer (mg/L), viable cell concentration (VCC; 10⁶ cells/mL), and cell viability (%) of the two groups were compared (Fig. 2).

### Design of N-1 intensification

Three distinct media, Media A (EX-CELL Advanced CHO) (Process Medium), Media B (BalanCD CHO Perfusion) (Process Medium), and Media C (OZBIO Perfusion) (Process Medium), were used in a comparative intensification study to improve cell growth during the N-1 phase. After achieving an adequate cell density on day 6, the cells were moved into the production phase. Media exchange was implemented starting on day 2 in increasing ratios (30%, 50%, 70%, and 100%) (Fig. 1b). Before returning the cultures to their original flasks, the exchange steps involved centrifuging them at 200 g for five minutes, removing the supernatant, and resuspending the cells in a new medium. To evaluate growth kinetics, viability (via) and viable cell concentration (VCC) were measured daily, starting on day 2.

### Fed-batch production configurations following N-1 intensification

Cells grown in Media A, B, and C during the N-1 phase were used for inoculation at 8 × 10⁶ cells/mL after their cell density was assessed at the end of day 6 (Fig. 1b). The initial cell density of 8 × 10⁶ cells/mL was established following preliminary optimization studies assessing various inoculation densities. The data from these preliminary studies are not included in this article. Two production configurations were evaluated. In the first configuration, cells cultured in Media A, B, or C during the N-1 phase were inoculated into the corresponding production medium (A→A, B→B, and C→C). In the second configuration, all N-1 groups were inoculated into a common production medium, Media A, to isolate the effect of N-1 physiological conditioning. Due to the high initial cell density, a fed-batch process was implemented with daily feedings of 4% Feed A and 0.4% Feed B, starting on day 1. During the fed-batch process, samples for metabolite analysis were collected after feeding, and glucose adjustment was performed based on the measured post-feeding glucose concentration. When the glucose concentration fell below 7 g/L, glucose was added to bring the culture medium up to 10 g/L. No additional glucose was added when the glucose concentration was above 7 g/L. VCC, cell viability, titer (mg/L), and total process duration (days) were used to compare the performance of cells grown under various N-1 conditions.

### Determination of cell viability and metabolites

Viable cell concentration and viability were determined using a Vi-CELL XR automated cell analyzer. Glucose, lactate, and ammonium concentrations were measured using the Nova Biomedical Bioprofile Flex2 device.

### Determination of titer

The calibration curve was established by preparing a series of dilutions from a reference monoclonal antibody solution (Reference Molecule, 25 mg/mL), with concentrations ranging from 0.02 to 0.4 mg/mL. Three distinct analysts prepared each concentration point on three separate days, injecting a volume of 50 µL. We plotted the peak areas against the amount of protein injected (µg) and used linear regression analysis. The regression equation is expressed as follows:

$$\:y=m.x+b$$

Where:

y = Peak area (AU), x = Injected protein amount (µg), m = Slope of the regression line, b = Intercept.

To demonstrate that the relationship was linear, the coefficient of determination (R²) needed to be a minimum of 0.995. Mobile Phase A was added to the test samples to achieve a concentration of 1 mg/mL, and then 10 µL volumes were injected. The peak area in the regression equation was used to determine the amount of protein injected, allowing for the calculation of concentration.

$$\:C=\frac{X}{{V}_{inj}}$$

Where:

C = Sample concentration (mg/mL), x = Calculated protein amount (µg), Vinj = Injection volume (µL).

A dilution factor (DF) was employed to determine the actual concentration, as follows:

$$\:{C}_{actual}=C.DF$$

This method has undergone evaluation for accuracy, repeatability, and system suitability. It enables reliable monitoring of mAb concentration during biopharmaceutical production.

### Ion exchange chromatography (IEX) analysis

Charge variants of monoclonal antibodies were analyzed by HPLC-based cation exchange chromatography. Separation was performed using a SEPAX Proteomix SCX-NP5 column under a linear salt gradient containing phosphate buffer (pH 6.0) and NaCl.

Samples were diluted with mobile phase and analysis was performed at 45 °C, at a flow rate of 0.35 mL/min, with UV detection at 214 nm.

Chromatograms were classified as acidic, main, and basic variants according to retention time and quantitatively evaluated based on relative peak area percentages.

### Statistical analysis

Statistical analyses of the acquired data were conducted utilizing GraphPad Prism (version 9.0, GraphPad Software, San Diego, CA, USA). A two-way analysis of variance (ANOVA) was used to assess variations across medium and time statistically. Significant differences among groups were evaluated using Tukey’s multiple comparison test. Results were presented as mean ± standard error (SEM), with *p* < 0.05 deemed statistically significant. Unless otherwise stated, n represents independent biological replicates corresponding to separate shake-flask cultures. Technical measurements were averaged before statistical analysis.

### Specific growth rate (µ) calculation

The specific growth rate (µ) was calculated using viable cell concentration (VCC) data measured at successive time points. The following equation was used in the calculations:

$$\:\mu\:=\:\frac{{lnX}_{2}-{lnX}_{1}}{{t}_{2}-{t}_{1}}$$

Here, Xt_1_ and Xt_2_ represents the viable cell densities (cells/mL) at the time points t_1_ and $\:{t}_{2}$ respectively. The specific growth rate was calculated for the time intervals between consecutive measurement days, and the results are reported in day⁻¹ units.

### Calculation of specific production (qP)

The specific production rate per cell (qP) was calculated as an interval-based productivity value using the increase in product concentration and the corresponding increase in integrated viable cell density (IVCD) over the same time interval. IVCD was first calculated using the trapezoidal integration method as follows:

$$\:IVCD=\sum\:\:\frac{{X}_{i}+{X}_{i+1}}{2}({t}_{i+1}-{t}_{i})$$

where X_i_ and X_i_₊₁ represent viable cell concentrations at two consecutive sampling points, and t_i_ and t_i_₊₁ represent the corresponding cultivation times.

The specific production rate (qP) was then calculated as:

$$\:{q}_{p}=\frac{{P}_{2}-{P}_{1}}{{IVCD}_{2}-\:{IVCD}_{1}}$$

where P₂ and P₁ represent the product concentrations at two consecutive sampling points, and IVCD₂ − IVCD₁ represents the integrated viable cell density accumulated over the same interval. Thus, the qP values reported in this study represent interval-based, daily cell-specific productivity values rather than cumulative productivity values. The calculated qP values were converted to pg/cell/day. For comparisons between LCD and HCD conditions, the reported qP values (~ 17 and ~ 31 pg/cell/day) represent the arithmetic mean of the interval-based daily qP values obtained throughout the evaluated production period.

## Results

### The impact of high seeding density on process efficiency and time

The initial phase of this study compared the effects of commencing the production phase at HCD and LCD on overall process efficiency, using the CHO-ZN cell line, which is commonly employed for the production of recombinant monoclonal IgG4 antibodies.

To that end, two distinct starting cultures were established at cell densities of 8 × 10⁶ cells/mL and 1.2 × 10⁶ cells/mL (HCD and LCD, respectively). Both groups were maintained in the same basal production medium and incubation conditions but were subjected to different feeding strategies. The LCD cultures were fed daily with 2% Feed A and 0.2% Feed B from days 1–4, followed by 2.5% Feed A and 0.25% Feed B from day 5 onward. In contrast, the HCD cultures received an intensified feeding regimen consisting of 4% Feed A and 0.4% Feed B daily from day 1 to support the higher initial biomass.

In contrast to fed-batch cultures initiated at traditional low cell density (LCD; 1.2 × 10⁶ cells/mL), this study showed that fed-batch cultures started at high cell density (HCD; 8 × 10⁶ cells/mL) improve process performance. Despite only being able to sustain high cell viability until day 7 (Fig. 2b), cultures seeded at HCD were able to achieve elevated titers of roughly 2500 mg/L during this brief period (Fig. 2c). However, the LCD group was able to sustain cell viability for a longer duration (Fig. 2b), the maximum titer that was attained was only approximately 1800–1900 mg/L (Fig. 2c). The higher initial cell density in the HCD group allowed the culture to reach maximum cell concentration earlier, but also rapidly shifted the culture toward nutrient, metabolic, and oxygen-transfer limitations (Fig. 2a). Even though an intensified feeding strategy (4% Feed A and 0.4% Feed B) was used from day 1 to support the high biomass, cell viability decreased below 70% by day 7, so the process had to be ended at that point.

The decline in viability observed in the HCD group was likely associated with the increased metabolic burden imposed by the higher initial biomass. Under high-seeding-density conditions, rapid nutrient consumption, accumulation of inhibitory byproducts, increased oxygen demand or oxygen-transfer limitation, and potential osmotic stress may collectively reduce culture stability. Similar reductions in culture stability under intensified high-cell-density conditions have previously been associated with nutrient depletion, accumulation of inhibitory metabolites, oxygen-transfer limitations, and osmotic stress in CHO cell culture systems [15, 33]. Although the intensified feeding strategy was applied to support the elevated biomass, the absence of comprehensive metabolite profiling, including amino acid consumption analysis and osmolality monitoring, prevents definitive identification of the dominant stress mechanism. Therefore, these explanations should be considered as plausible hypotheses rather than direct mechanistic conclusions.

The HCD process ended on day 7, but the titer reached roughly 2500 mg/L in that short time, indicating a substantial reduction in culture duration without compromising yield (Fig. 2c). These results suggest that the HCD technique can be utilized to achieve increased product output in a shorter production period. Similar reductions in fed-batch duration using high-seeding-density strategies have also been reported previously in intensified upstream processes [15].

The differences in titers observed were likely associated with the higher total cell biomass at the start of the production phase, together with differences in interval-based qP and culture-phase dynamics. Moreover, the average interval-based qP values, calculated from daily qP profiles, were markedly higher under high cell density (HCD) conditions (~ 31 pg/cell/day) compared with low cell density (LCD) conditions (~ 17 pg/cell/day). The increased productivity observed under HCD conditions was associated not only with elevated biomass but also with higher interval-based qP values. Previous studies have shown that higher initial cell densities can increase total protein synthesis capacity during the early production phase, even when cell-specific productivity remains unchanged. Therefore, the higher titers observed under HCD conditions may reflect the combined contribution of increased initial biomass, altered culture-phase dynamics, and elevated interval-based qP values [33]. Increased seeding densities may activate metabolic pathways associated with production and influence the regulation of the cell cycle and gene transcription [34, 35].

Similar results have been reported in the literature, which supports these findings. Stepper et al. (2020) demonstrated that in fed-batch production processes using CHO cells, starting cultures at a cell density of 7–10 × 10⁶ cells/mL resulted in a 30–50% increase in titer and a reduction of 2–3 days in process duration compared to cultures initiated at lower densities. This may improve facility utilization by shortening effective production time.

Xu et al. (2020) noted that although high seeding densities accelerate the accumulation of early-phase titers, the subsequent decline in cell viability must be carefully managed to maintain process robustness. A comparable pattern was observed in the present study. In the baseline HCD fed-batch configuration without N-1 media-exchange intensification, the maximum titer was approximately 58% higher than that of the LCD group (Fig. 2c). The results indicate that under HCD conditions, the production process exhibits increased time efficiency, facilitating a higher number of production cycles within a specified timeframe. Notably, both groups achieved identical maximum cell densities, which is unexpected, as it suggests that cell counts alone do not account for the variations in titer. Instead, they likely reflect the effects of the metabolic and environmental conditions experienced by the cells during the production phase.

Additionally, the HCD group’s early feeding strategy (Feed A and B) was sufficient to meet the initial needs of the cells; however, the fact that viability dropped over time suggests that more advanced feeding and control methods may be necessary to maintain the culture’s performance over time.

In summary, fed-batch processes that begin with a high cell density can significantly enhance overall production performance by achieving elevated titers in reduced timeframes, particularly when integrated with appropriate feeding and environmental control methodologies. These findings suggest that high cell density-based approaches may support the development of intensified upstream biopharmaceutical manufacturing strategies; however, additional validation in controlled bioreactor systems is required before industrial implementation.

### Impact of media exchange-based process intensification on cell density and viability in the N-1 stage

In the second part of this project, a strategy for process intensification was developed to maintain the efficiency improvements achieved by starting the fed-batch process at a higher cell density. The effects of various commercially available media formulations (henceforth referred to as Media A, Media B, and Media C) on cell growth were compared to determine which medium provided the highest cell biomass advantage.

**Fig. 3:**
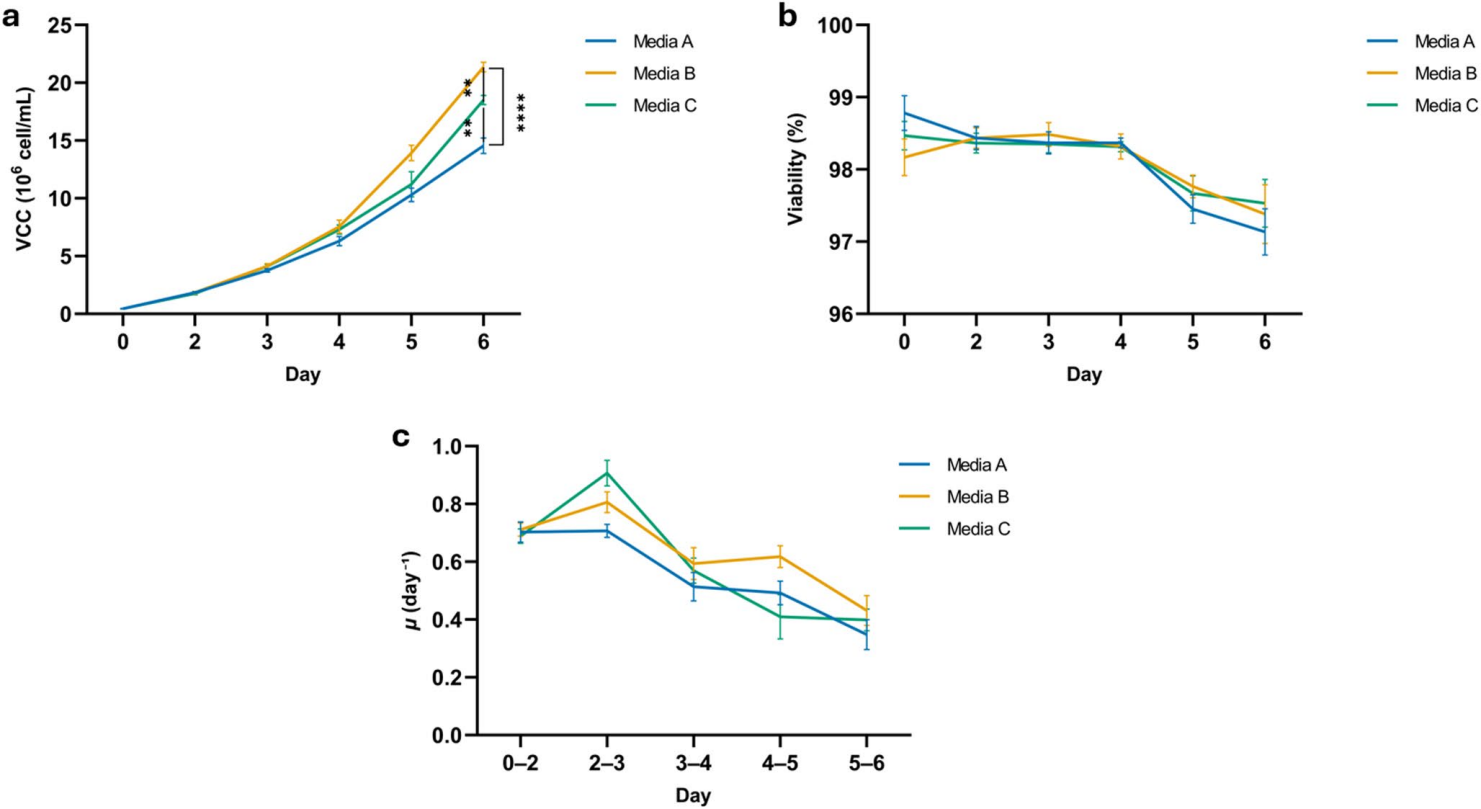
Viable cell concentration (VCC) and viability profiles of CHO cells cultivated in three media (Media A, B, and C, shown with blue, yellow and green, respectively). (A) VCC trends over six days show that distinct growth profiles were observed between media conditions (*p* < 0.001). (B) Viability remained stable (97–99%) across all groups, with no significant differences (*p* > 0.05). (C) Specific growth rate (µ) of CHO cells in different media (A, B, and C), calculated from VCC between consecutive time intervals. The data are shown as mean ± SEM (*n* = 6)

A medium exchange protocol was implemented on the second day of the N-1 phase, with exchange ratios increasing in increments of 30%, 50%, 70%, and 100% (Fig. 1b). Cells were centrifuged at 200 g for 5 min and separated from the existing medium at each step. Then they were returned to the target medium. This approach enabled gradual adaptation of the cells to the target N-1 medium while minimizing abrupt medium-composition changes. Upon achieving the appropriate cell density by day 6, the cells were transitioned to the production phase. The study monitored cell growth rate and viability to assess the impact of medium selection on bioprocess performance (Fig. 3).

Our results showed that efficiency gains are achievable by initiating fed-batch processes at elevated cell densities. Nonetheless, sufficient cell biomass at the N-1 stage is essential for realizing these advantages in the production phase. Achieving such densities typically requires multiple seed expansion steps through conventional seed train methods, which may increase process duration and resource consumption. To achieve process intensification, media exchange was implemented during the N-1 phase of this study, utilizing various commercial media formulations (Media A, B, and C).

At the end of day 6, the viable cell concentrations were 21.3 × 10⁶ cells/mL for cultures produced in Media B, 18.5 × 10⁶ cells/mL for Media C, and 14.5 × 10⁶ cells/mL for Media A (Fig. 3a). These results show that the choice of culture medium in the N-1 phase has a significant impact on cell proliferation and may significantly influence process intensification performance. The Media B group achieved a cell density that was approximately 47% higher than Media A, while the Media C group showed an increase of about 28% higher than Media A. Notably, Media B maintained a sustained increase in viable cell concentration after day 4, although the specific growth rate gradually decreased during the later culture phase. This pattern suggests that perfusion-oriented formulations, such as Media B, offer benefits in terms of nutrient availability and sustained cell proliferation. Throughout the 6-day culture period, cell viability consistently remained elevated (97–99%) across all media conditions (Fig. 3b). These results indicate that the cells tolerated the implemented media exchange regimen, including centrifugation and resuspension steps, without substantial loss of viability.

According to the cell growth curves, it was observed that under Media B and C conditions, the cells showed a rapid increase in the early culture phase, and the growth rate decreased in the following days. The specific growth rate (µ) data presented in Fig. 3c also supports this, showing that the growth rate reached its maximum between days 2–3 and then gradually decreased. This indicates that the cells transitioned from the exponential phase to the stationary phase.

By day 6, it was determined that the cells had reached a high cell density and maintained their viability, indicating that the cells were suitable for transition into the production phase. Accordingly, the inoculation procedure was performed on day 6.

### Effects on fed-batch production of various N-1 conditions attained by process intensification

To distinguish between the combined effect of N-1 and production media and the specific carry-over effect of N-1 conditioning, two experimental configurations were evaluated. In the first configuration, cells expanded in Media A, B, or C during the N-1 phase were transferred into the same corresponding production medium. In the second configuration, all N-1 groups were transferred into a common production medium, Media A, thereby isolating the effect of N-1-derived physiological conditioning on subsequent fed-batch performance.

In this study, cells initially grown in different media (Media A, B, and C) during the N-1 phase were inoculated using the same media (A→A, B→B, and C→C) during the transition to the production phase.

**Fig. 4:**
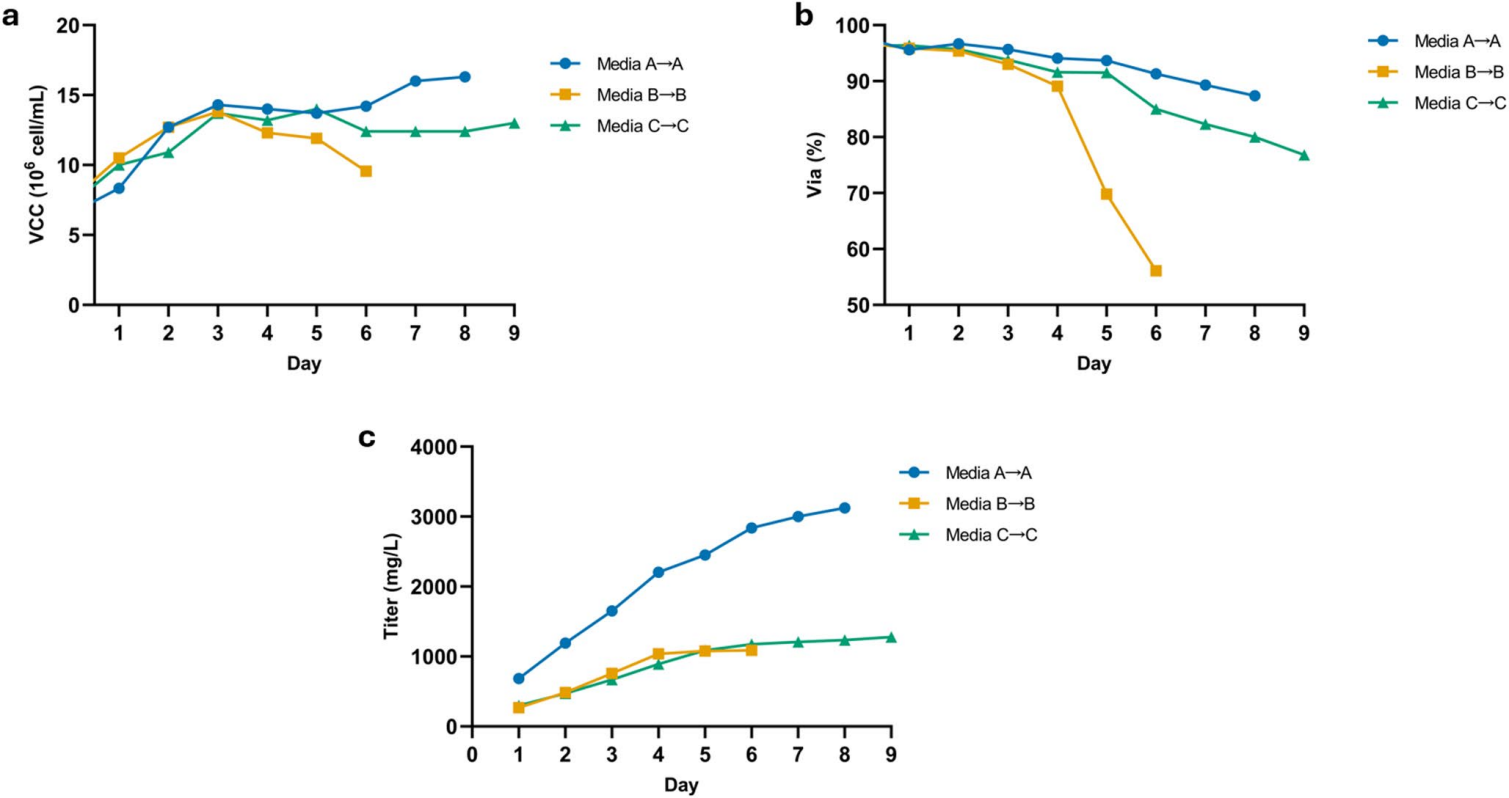
Fed-batch production performance of cells cultured in various media during the N-1 phase and subsequently inoculated into the corresponding media during the production phase (A→A, B→B, and C→C). (A) Viable cell concentration (VCC) profiles throughout the culture period. (B) Profiles of cell viability (%). (C) Profiles of titer (mg/L). The data are shown as mean ± SEM (*n* = 3)

According to the data presented in Fig. 4, cell density initially increased in all groups, but differentiated growth profiles emerged over time (Fig. 4a). In the Media A→A condition, cell density continued to increase throughout the culture period, while in the Media B→B group, cell density decreased earlier in the phase. In contrast, cells in the Media C→C group entered the stationary phase after reaching a plateau.

When viability data were examined (Fig. 4b), it was observed that cell viability remained more stable in the Media A→A group, while a significant loss of viability was observed in the Media B→B group, especially in the later days. In the Media C→C group, viability values were maintained at a moderate level.

When product concentration results (Fig. 4c) were evaluated, it was seen that the Media A→A condition provided significantly higher production compared to the other groups. In the Media B→B and Media C→C groups, a lower and more limited production profile was observed.

After that, cells grown in different media during the N-1 phase were transferred into a common production medium (Media A). This configuration enabled isolation of the physiological carry-over effects originating from N-1 conditioning. In contrast to the prior study, the production medium variable was removed, facilitating the evaluation of effects exclusively attributable to the N-1 phase. This experimental design enabled the examination of the influence of N-1 media on cell proliferation and the continuation of these effects into the production phase, termed the “N-1 carry-over effect.” Previous intensified fed-batch studies similarly demonstrated that seed-stage process conditions can significantly affect subsequent production performance even when production-phase conditions are maintained similarly [3].

The N-1 carry-over effect occurs when cellular conditions established during the seed expansion phase (N-1) persist throughout the production phase, influencing the performance of subsequent processes. This phenomenon may subsequently affect cell growth, productivity, metabolic behavior, and product quality during fed-batch production. This encompasses alterations in cellular metabolism, stress response, and productivity influenced by cultural factors such as medium composition, cell-specific perfusion rate (CSPR), and seeding density. Schulze et al. (2021) demonstrated that regulating CSPR during perfusion-based N-1 culture resulted in enhanced inoculum quality and increased growth and productivity in the production bioreactor [36, 37]. Xu et al. (2020) demonstrated that intensification strategies applied during N-1, even in non-perfusion formats, led to increased titers and reduced culture durations in the fed-batch stage, supporting the concept of seed-stage physiological conditioning [3].

**Fig. 5:**
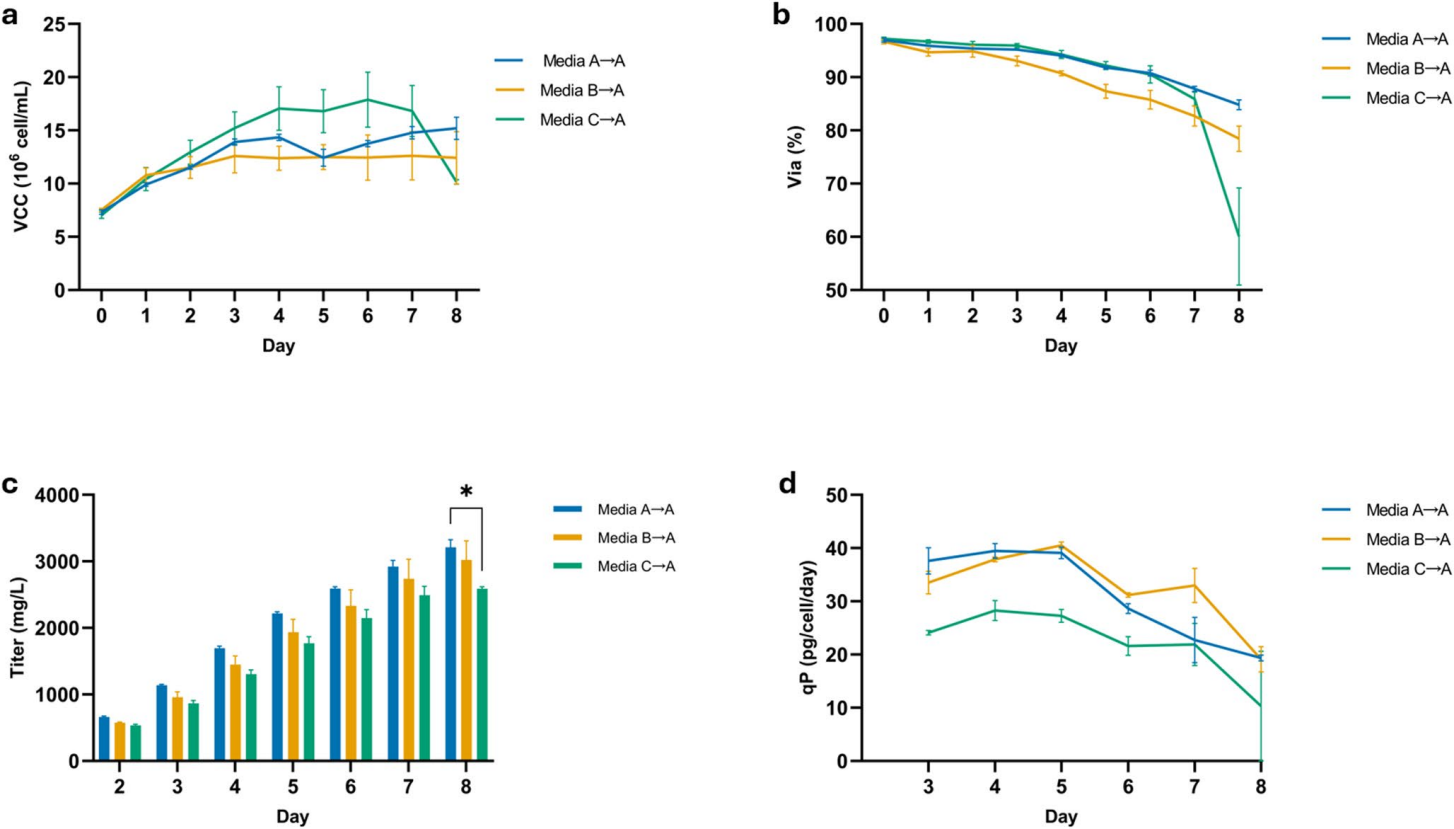
The growth, viability, and titer profiles of CHO cells cultured in three different media (A, B, and C) during fed-batch production. (A) Viable cell concentration (VCC) profiles over eight days of cultivation. (B) Cell viability profiles during fed-batch cultivation; viability remained relatively high but showed condition-dependent decreases over time. (C) Titer profiles showing increased mAb accumulation in the Media A→A condition compared with the Media B→A and Media C→A conditions. (D) Interval-based daily specific productivity profiles (qP, pg/cell/day). The data are presented as mean ± SEM (*n* = 4). (*p < 0.05)

Figure 5a shows that cell density (VCC) increased over time in all groups. Group C→A showed faster growth in the early phase, while Group A→A showed a more balanced increase. Group B→A had lower VCC values compared to the other groups.

Figure 5b shows that viability decreased over time in all groups. Group A→A maintained more stable viability, while Group B→A and especially Group C→A showed more significant decreases.

As shown in Fig. 5c, titer values increased over time in all groups during the production phase. However, it was observed that cells cultured under Media A→A conditions reached higher titer values compared to other groups. Specifically, a maximum titer of approximately 3200–3300 mg/L was obtained in the Media A→A group on day 8, while this value remained around 3000 mg/L in the Media B→A group, and a lower production performance of approximately 2500–2600 mg/L was observed in the Media C→A group.

Despite the use of a common production medium, differences between the groups persisted throughout the production phase. The fact that cells grown in Media A reached higher titer values and maintained their viability for a longer period in the production phase suggests that the cells may have entered the production phase in a more favorable physiological state. This observation is consistent with the concept of the “N-1 carry-over effect” described in the literature [38, 39].

The interval-based daily qP data presented in Fig. 5d suggest that the observed differences were not solely related to total biomass accumulation but were also associated with differences in apparent cell-specific productivity. Higher qP values are observed in Media A→A and Media B→A groups, while lower qP is seen in the Media C→A group. The higher qP values obtained in the Media A→A group, particularly in the early production phase (days 3–5), suggest that this group exhibited more favorable production behavior during the early production phase.

In contrast, the low qP values observed in the Media C→A group and the significant decrease in the following days suggest that the cells exhibited less favorable production behavior and metabolic characteristics during production. Therefore, N-1 medium composition appears to affect not only cell growth, but also apparent cell-specific production behavior during the subsequent production phase.

**Fig. 6:**
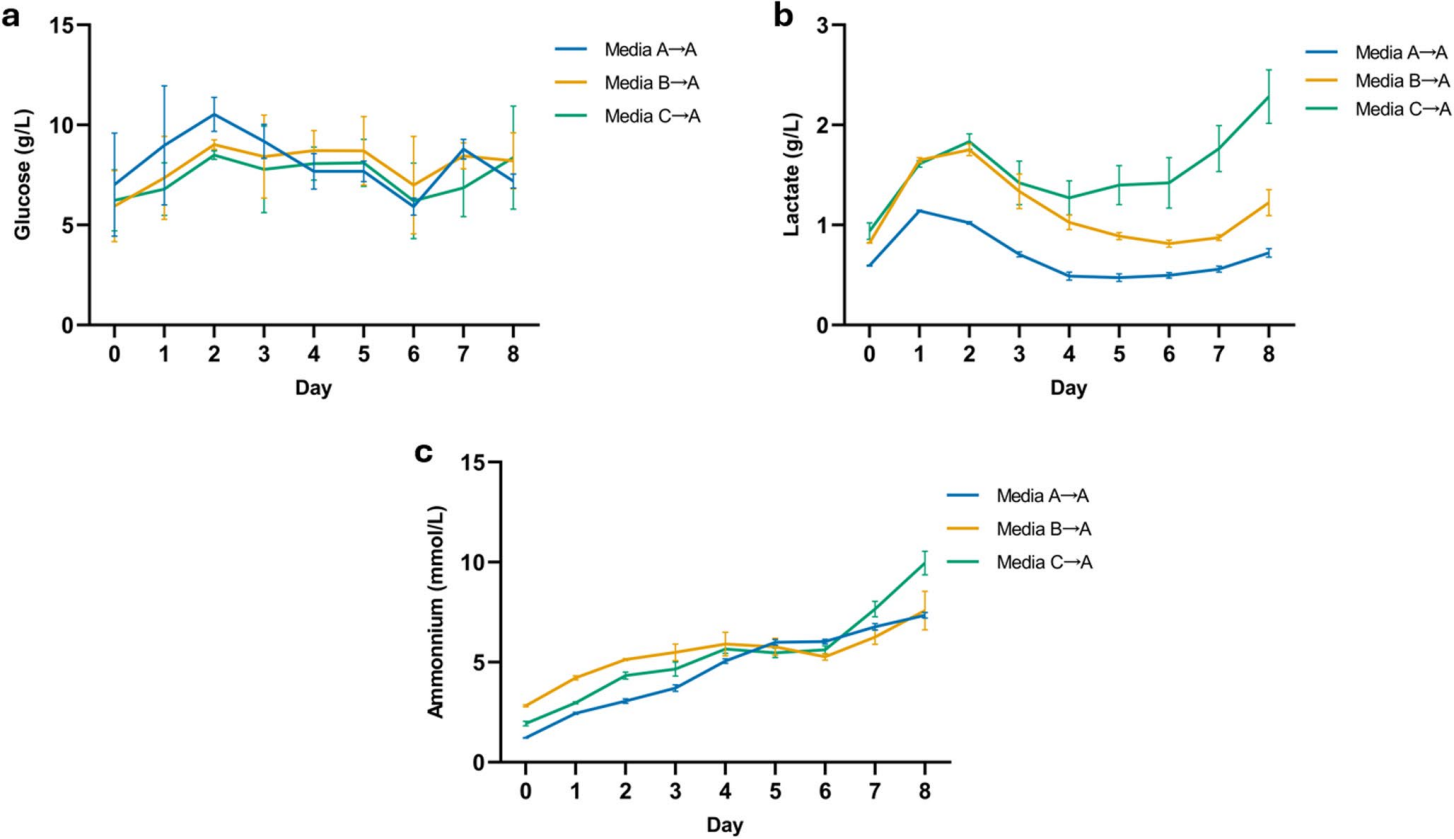
Metabolite profiles of fed-batch cultures inoculated into a shared production medium (Media A) following cultivation in various N-1 media. (A) Glucose concentration profile (g/L). (B) Lactate concentration profile (g/L). (C) Ammonium concentration profile (mmol/L). The data are shown as mean ± SEM (*n* = 4)

Glucose concentrations remained relatively stable and comparable across all groups throughout the culture period (Fig. 6a).

Lactate accumulation increased progressively during cultivation and reached the highest levels under the Media C→A condition (Fig. 6b). In contrast, lactate accumulation was more limited in the Media A→A group.

Ammonium levels increased in all cultures but remained lower in the Media A→A group (Fig. 6c). The lower lactate and ammonium accumulation observed in the Media A→A condition was consistent with its higher qP and improved viability profile, suggesting a more favorable metabolic state during production. In contrast, the increased lactate and ammonium accumulation in Media C→A may indicate less efficient carbon and nitrogen metabolism, which could contribute to reduced productivity and faster viability decline (Table 1).

**Table 1:** Charge variant distribution of monoclonal antibodies generated under varying culture conditions

Condition	Acidic variants (%)	Main peak (%)	Basic variants (%)
Reference	18.35	58.32	23.32
Media A→A	28.89	57.96	13.16
Media B→A	23.44	60.99	15.57
Media C→A	21.61	64.41	13.98

Values show the percentage of charge variants that ion exchange chromatography found. The data are shown as the average of four separate tests (*n* = 4).

When the ion exchange chromatography results were examined, it was observed that the charge variant distributions differed from the reference product in all groups. It is noteworthy that the proportion of the acidic variant increased significantly in the Media A→A group (28.9%), while the proportion of the basic variants decreased (13.2%). In the Media B→A and Media C→A groups, the proportions of the acidic variants were 23.4% and 21.6%, respectively, which were lower than Media A→A but still higher than the reference product.

When the main peak ratio was examined, it was observed that the Media C→A group had the highest main peak percentage (64.4%), followed by the Media B→A (61.0%) and Media A→A (58.0%) groups. Compared to the main peak ratio of the reference product (58.3%), higher main peak values were obtained, especially under the Media B→A and Media C→A conditions.

The charge variant data further indicate that N-1 and production-phase medium conditions may influence product quality attributes in addition to cell growth and productivity. In particular, the increased acidic variant ratio under the Media A→A condition suggests that culture conditions may have altered product charge heterogeneity, potentially through changes in post-translational modification patterns or degradation-related modifications.

Collectively, the standardized production-medium experiments support the presence of an N-1 carry-over effect. Differences in qP, metabolite accumulation, viability, and titer persisted depending on N-1 medium history, indicating that seed-stage physiological conditioning substantially influenced subsequent fed-batch behavior. In particular, the more balanced metabolite profile, higher production per cell (qP), and superior titer values observed in the Media A→A condition suggest that this medium may support a physiological state more favorable for fed-batch production. In contrast, the lower qP and production performance observed in the Media C→A condition, along with increased lactate and ammonium accumulation, reveal that the cells exhibit a less favorable metabolic behavior.

## Discussion

This study systematically evaluated the effects of medium optimization and media exchange strategy applied during the N-1 phase in the CHO-ZN cell line on fed-batch production processes initiated with high cell density (HCD). Previous studies have investigated intensified upstream processing, but the combined influence of N-1 medium composition and high-seeding-density inoculation remains insufficiently characterized. In this respect, the study provides additional insight into the relationship between N-1 physiological conditioning and subsequent fed-batch production performance. The findings showed that under intensified N-1 media-exchange conditions combined with HCD inoculation, the production phase was shortened by approximately 36% and maximum titer values reached levels of ~ 3500 mg/L. In contrast, in conventional low cell density (LCD; 1.2 × 10⁶ cells/mL) fed-batch cultures, the maximum titer remained at ~ 1850 mg/L, corresponding to approximately 53% lower titer values compared to intensified HCD cultures. Average interval-based qP analyses showed that while levels of approximately 31 pg/cell/day were reached under HCD conditions, this value remained around 17 pg/cell/day under LCD conditions. These findings suggest that the increase in titer was associated not only with increased cell biomass but also with higher average interval-based qP values during the evaluated production period. Because the reported qP values were calculated as the arithmetic mean of interval-based daily qP measurements, differences in culture-phase distribution between LCD and HCD cultures may also contribute to the observed productivity differences. HCD cultures reached the plateau/stationary phase earlier, whereas LCD cultures remained in the growth phase for a longer proportion of the culture duration. Accordingly, the observed qP differences likely reflect both altered culture dynamics and differences in apparent production behavior during the evaluated production period. Because different feeding strategies were applied to LCD and HCD cultures to accommodate their distinct biomass demands, the observed differences cannot be attributed exclusively to seeding density. Rather, they reflect the combined effect of high initial cell density and intensified feeding conditions. Future studies using matched or feedback-controlled feeding strategies will be required to isolate the independent contribution of initial seeding density from feeding-related effects. Rather than proposing a directly scalable industrial intensification platform, this study aimed to investigate how N-1 medium composition and physiological conditioning influence subsequent production performance under high-seeding-density fed-batch conditions.

The media exchange strategy applied during the N-1 phase significantly increased cell density, simplifying the seed train structure. While the conventional passaging approach reached levels of approximately 3 × 10⁶ cells/mL by the end of day 3, media exchange resulted in cell densities ranging from 15 to 22 × 10⁶ cells/mL by the end of day 6. This allowed for direct inoculation into the production phase with 8 × 10⁶ cells/mL, eliminating at least one additional growth step. Considering the time saving of approximately 3–4 days achieved in the N-1 phase, along with the reduction in production phase time, a potential reduction of approximately 4–6 days in total process time emerges. However, this total saving may vary depending on the seed train strategy used. This time advantage can be further increased, especially when using intensified N-1 platforms (continuous or semi-continuous systems). Furthermore, starting production with a higher and more homogeneous cell population enables more standardized production-phase inoculation conditions, reducing variation and lowering operational complexity.

When the effects of the culture media used in the N-1 phase on cell growth were examined, Media B and Media C reached levels of approximately 21 × 10⁶ cells/mL and 18 × 10⁶ cells/mL, respectively, while in Media A this value remained around 14–15 × 10⁶ cells/mL. However, the highest titer (~ 3200–3300 mg/L) and the highest qP values in the production phase were obtained under the Media A→A condition. This indicates that N-1 cell density alone is not sufficient to predict subsequent production performance and that the physiological state established during the N-1 phase is also an important determinant of fed-batch outcome. Similar observations were reported by Yongky et al. [3], where substantially higher N-1 viable cell concentrations achieved through perfusion-based seed intensification did not result in proportional increases in final production titer. This observation is consistent with previous reports suggesting that conditions favoring rapid cell expansion may not necessarily support optimal recombinant protein production performance [15]. When comparing the conditions where the same culture medium was used in the N-1 and production phases with the conditions where all groups were transferred to Media A in the production phase, it was observed that the production culture medium had a strong influence on cell performance. The Media A→A condition maintained higher viability and achieved the highest titer values during production. When all groups were transferred into Media A during production, differences in qP, metabolite accumulation, viability, and titer still persisted depending on the N-1 medium history. This supports the presence of an N-1 carry-over effect, indicating that production outcome is influenced not only by production-medium composition, but also by the physiological state established during the N-1 phase.

Comparing all groups in Media A during the production phase further supported the concept of the “N-1 carry-over effect”. Lower lactate and ammonium accumulation was observed in the Media A→A group, while significantly higher levels of these metabolites were determined in the Media C→A group. Previous intensified seed train studies have similarly emphasized the importance of controlling nutrient balance and waste metabolite accumulation prior to production inoculation [15]. These metabolic differences are consistent with qP data and suggest that physiological characteristics established during the N-1 phase may persist into the production phase. Product quality analyses were consistent with these observations. The acidic variant rate increased to 28.9% in the Media A→A condition, while this value was determined as 18.3% in the reference product. In contrast, the main peak rate reached its highest level at 64.4% in the Media C→A group. These findings show that a balance should be established between high production performance and product quality characteristics. In the literature, it has been shown that temperature shifting strategies applied in the production phase can reduce acidic variant formation and improve the charge-variant distribution [40, 41]. Therefore, it is recommended to optimize temperature, pH and feeding strategies together. A significant limitation of this study is that the experiments were conducted on a shake flask scale. Therefore, the results obtained need to be validated in a bioreactor environment. In industrial applications, intensified N-1 operations are commonly implemented using perfusion-based systems such as ATF or TFF platforms [15]. Controlling pH, dissolved oxygen, and CO₂ is particularly critical in high cell density systems. Although the stepwise centrifugation-based media exchange strategy used in this study was effective for evaluating N-1 physiological conditioning at shake flask scale, it is not directly transferable to large-scale manufacturing. Industrial implementation of such an approach would likely require adaptation to scalable and closed cell-retention systems, such as ATF- or TFF-based perfusion or semi-continuous media exchange platforms. Nevertheless, the findings demonstrate that modulation of the N-1 cellular environment alone can substantially influence subsequent production performance, supporting the importance of seed-stage physiological conditioning during intensified upstream processing [1, 10, 26]. However, the molecular mechanisms underlying this carry-over effect remain to be fully elucidated. Additional analyses, including amino acid profiling, osmolality monitoring, transcriptomic assessment, and intracellular metabolic characterization, would be required to define the mechanistic basis of the observed differences.

In conclusion, this study demonstrates that N-1 medium composition influences not only biomass expansion but also subsequent fed-batch productivity, metabolic behavior, and charge variant distribution under high-seeding-density conditions. The findings highlight the importance of N-1 physiological conditioning during intensified upstream processing and further support previous reports describing the potential of intensified non-perfusion seed strategies for improving fed-batch manufacturing performance [3].

Although the centrifugation-based medium-exchange approach used here is not directly scalable, the results demonstrate that modulation of the N-1 cellular environment can substantially affect subsequent fed-batch productivity, metabolic behavior, and charge variant distribution. Further validation in controlled bioreactor systems, preferably using scalable ATF- or TFF-based N-1 intensification platforms, together with additional mechanistic analyses, will be required before industrial implementation.

## Data Availability

The data that support the findings of this study are available from the corresponding author upon reasonable request.

## Abbreviations

mAb / mAbs: monoclonal antibody / monoclonal antibodies
CHO: Chinese Hamster Ovary
CHO-ZN: Chinese Hamster Ovary – ZN subline
HCD: high cell density
LCD: low cell density
N-1: seed train phase prior to production (one phase before N phase)
VCC: viable cell concentration
Via: viability
qP: specific productivity
